# Psychological distress in adolescence and later economic and health outcomes in the United States population: A retrospective and modeling study

**DOI:** 10.1371/journal.pmed.1004506

**Published:** 2025-01-16

**Authors:** Nathaniel Z. Counts, Noemi Kreif, Timothy B. Creedon, David E. Bloom

**Affiliations:** 1 The Kennedy Forum, Brigantine, New Jersey, United States of America; 2 Department of Health Policy and Management, Columbia University Mailman School of Public Health, New York, New York, United States of America; 3 Department of Pharmacy, University of Washington School of Pharmacy, Seattle, Washington, United States of America; 4 Office of the Assistant Secretary for Planning and Evaluation, U.S. Department of Health and Human Services, Washington, District of Columbia, United States of America; 5 Department of Global Health and Population, Harvard T.H. Chan School of Public Health, Boston, Massachusetts, United States of America; Massachusetts General Hospital, UNITED STATES OF AMERICA

## Abstract

**Background:**

Federal policy impact analyses in the United States do not incorporate the potential economic benefits of adolescent mental health policies. Understanding the extent to which economic benefits may offset policy costs would support more effective policymaking. This study estimates the relationship between adolescent psychological distress and later health and economic outcomes and uses these estimates to determine the potential economic effects of a hypothetical policy.

**Methods and findings:**

This analysis estimated the relationship between psychological distress in those aged 15 to 17 years in 2000 and economic and health outcomes approximately 10 years later, accounting for an array of explanatory variables using machine learning–enabled methods. The cohort was from the National Longitudinal Study of Youth 1997 and nationally representative of those aged 12 to 18 years in 1997. The cohort included 3,343 individuals under age 18 years in round 4 who completed the Mental Health Inventory-5 (MHI-5). Round 1 captured 50 explanatory variables that covered domains of potential confounders, including basic demographics, neighborhood environment, family resources, family processes, physical health, school quality, and academic skills. The exposure included a binary variable of clinically significant psychological distress (MHI-5 score of less than or equal to 3) and a categorical variable of symptom severity on the MHI-5. Outcomes covered domains of employment, income, total assets at age 30 years, education, and health approximately 10 years later.

Forty-seven percent of the cohort were black and Hispanic, and 4.4% had past-month clinically significant psychological distress. Past-month clinically significant psychological distress in adolescence led to a 6-percentage-point (95% confidence interval [CI] [−0.08, −0.03]) reduction in past-year labor force participation 10 years later and $5,658 (95% CI [−6,772, −4,545]) USD fewer past-year wages earned. We used these results to model the labor market impacts of a hypothetical policy that expanded access to mental health preventive care and reached 10% of youth who would have otherwise developed clinically significant psychological distress. We found that the hypothetical policy could lead to $52 (95% credible interval [51,54]) billion USD in federal budget benefits over 10 years from labor supply impacts alone. This study faced limitations, including potential unmeasured confounding, missing data, and challenges to generalizability.

**Conclusions:**

Our findings showed the impacts of adolescent mental health policies on the federal budget and found potentially large effects on the economy if policies achieve population-level change.

## Abstract 2

Nathaniel Counts and colleagues estimate the relationship between adolescent psychological distress and later health and economic outcomes, and use these estimates to determine the potential economic impact of a hypothetical policy.

## Author summary

**Why was this study done?:**

- A large literature identifies ways in which improving adolescent mental health yields long-term economic benefits, from greater labor force participation to lower public benefit utilization.
- Government budget analysts do not reflect these benefits in their analyses because they lack the resources to comprehensively model every domain of policy, and the existing literature does not offer parameters that work well with their modeling approaches.
- This study offers parameters that could fit into budget impact models and assesses the importance of including these parameters to the model outputs.

**What did the researchers do and find?:**

- We estimated the relationships between psychological distress in adolescence and outcomes 10 years later, accounting for several other factors, in a nationally representative longitudinal cohort.
- We applied these estimated relationships in the context of a simplified budget impact model with a hypothetical policy to assess the magnitude of the budget effects of improving adolescent mental health, based on changes in labor force participation.
- We found that psychological distress is related to later economic outcomes, such as less annual labor force participation 10 years later, and that the hypothetical policy could yield billions of dollars of budgetary benefits from increased labor force participation alone.

**What do these findings mean?:**

- Government budget analysts can use these estimated relationships between psychological distress in adolescence and later outcomes as parameters in their models to assess the impacts of policies that improve adolescent mental health and doing so would meaningfully affect their model outputs.
- This study had limitations, such as potentially unobserved confounding in the parameter estimates and simplifying assumptions in the budget estimates.
- Improving budget impact analysis could enable greater public investment in adolescent mental health and address the current crisis in youth mental health facing the United States.

## Introduction

Over the past decade, adolescent mental health in the United States declined substantially, which was further exacerbated by the COVID-19 pandemic [1–3]. The trend likely reflects the impacts of shifting social, technological, political, and economic forces on the lives of children, which may progress in the future as these forces further evolve [4–6]. Declining mental health in adolescence may have life-course impacts, which could lead to worse long-term outcomes if actions are not taken to mitigate potential consequences [7]. National policy reforms and investments will likely be needed to reverse the current trend and ultimately improve adolescent (and life-course) mental health in the United States.

Unfortunately, public action on the necessary scale faces structural difficulties, given the realities of the federal budgeting process. Although policies that improve adolescent mental health may also confer some offsetting budget returns [8], the policy impact analyses produced by budget entities such as the Congressional Budget Office (CBO) rarely include these effects [9]. Without accounting for later returns, investments in adolescent mental health would be considered to increase the deficit and policymaking may be limited to small pilots and demonstration programs.

Policy impact analyses do not count potential offsetting returns from investments in adolescent mental health primarily due to resource constraints [9]. For budget entities, accurate parametrization is also difficult due to measurement issues and a high degree of confounding in the relationship between adolescent mental health challenges and later economic outcomes. The literature offers many important estimates, but it does not provide the full array of parameters necessary for modeling the budget effects of improving adolescent mental health in existing budget models. The evidence that the potential policy impacts are substantial enough to be worth estimating is also scant, making it less of a priority for budget entities in developing comprehensive models.

The current literature indicates that adolescent mental health can influence later economic outcomes in 2 ways: through health selection or social causation [10]. Health selection theory posits that an individual’s experience of challenges to cognitive, affective, social, and physical functioning causes them to select into labor market roles that reflect these difficulties, explaining the relationship between adolescent mental health and later outcomes (sometimes also referred to as “social drift” in the mental health literature) [11]. Social causation theory posits that exogenous forces such as socioeconomic circumstances and other stressors influence both mental health and economic outcomes, as adversity harms wellbeing and labor market potential. Reconciling these 2 theories, economists have proposed production functions for human capital development that consider initial endowments and investment from family and the broader community at each stage of development, accounting for observed and unobserved confounders [12]. Empirical studies find relationships between adolescent mental health and different later economic outcomes [13–15], with both health selection and social causation likely playing a role in these relationships [11,16,17]. The available literature does not comprehensively estimate all of the relationships needed for policy impact modeling in a single study. Some studies also face likely unmeasured confounding, strong assumptions about model specifications that may not be justified, or threats to the generalizability of the findings to the United States population as a whole, which may undermine the use of their findings in policy impact modeling.

Other theoretical literature examines the ways in which adolescent mental health challenges may have consequential macroeconomic and federal budget impacts [18–20]. Considering a standard production function that relates the contributions of labor, capital, and total factor productivity to overall output (i.e., gross domestic product), mental health conceptually relates to all three of these inputs. Individuals who face mental health challenges are likely to supply less labor. Depending on how the human capital component is incorporated into the production function, adolescent mental health challenges can reduce educational attainment and/or time in the labor force, both of which relate to measures of worker skill. Finally, individuals who face mental health challenges are also less likely to save money, diminishing the capital supply, and may also be less able to contribute to the creation of new sources of capital, such as valuable intellectual property [21]. All of these impacts decrease potential federal tax revenue and may increase federal spending if more individuals require income supports [22]. The macroeconomic impacts may also prompt federal monetary policy actions that increase the interest rate paid on federal debt, in addition to increases in the debt itself [23]. Few studies empirically examine the relationship between mental health and macroeconomic growth [24], while more studies estimate the economic effects of specific interventions to address adolescent mental health challenges, with most focusing on savings in particular areas of public spending rather than the full range of secondary macroeconomic effects that impact the federal budget [25,26].

This paper aims to address identified gaps in the literature by first comprehensively estimating parameters for the relationship between clinically significant psychological distress in adolescence and later labor and health outcomes, which budget analysts could incorporate into existing economic models. We then offer an exploratory analysis of the extent to which a hypothetical policy—scaling up integrated mental health preventive interventions in primary care for adolescents—might have important impacts on the macroeconomy and federal budget, indicating whether incorporating new variables in economic models may be worthwhile for budget entities. To the extent that the budget benefits partially offset the costs of proposed policies, more accurate modeling may facilitate necessary policy action to address the adolescent mental health crisis in the United States.

## Methods

### Ethics statement

The human subjects research in this study were limited to secondary analysis of publicly available and de-identified data and therefore did not require ethical approval. The study was conducted in accordance with the Declaration of Helsinki.

### Estimating the relationship between adolescent psychological distress and later outcomes

#### Data and cohort

Our data came from the National Longitudinal Survey of Youth 1997 (NLSY97), an annually administered panel survey of 8,984 people aged 12 to 18 years at first survey administration in 1997 [27]. This initial cohort comprised a nationally representative cohort of 6,748 participants and a supplemental cohort of 2,236 participants designed to oversample black and Hispanic respondents, both of which were included in this study. NLSY97 had an overall retention rate of 83.2% across the 2 combined cohorts by survey administration round 14, the round from which we took all of our later outcomes, except for assets at age 30 years, as not all individuals turned 30 years on the same round. Our study cohort included NLSY97 participants who were interviewed in round 4 in 2000—the round when individuals first completed the Mental Health Inventory-5 (MHI-5)—who were aged 17 years or younger, and who completed all 3 questions in the psychological distress subscale of the MHI-5.

#### Exposure

Table 1 summarizes the variables of interest. Our exposure variable came from the MHI-5 in survey round 4. The MHI-5 included 5 items on a four-point Likert scale assessing past-month mood based on the presence of psychological wellbeing and the absence of psychological distress [28]. We used the three-item psychological distress subscale of the MHI-5 in which scores range from 0 to 9, with higher scores indicating less distress. The psychological distress subscale was the only scale or subscale of the MHI-5 that was validated as predictive of clinically significant anxious or depressive symptoms and diagnosable anxiety and depression disorders at a score of 3 or less [28]. We tested a dichotomous variable of those with a score of less than or equal to 3 and those with a score of 4 or greater as well as a categorical variable of population mean psychological distress scores at values of 6, 7, and 8. The mean score in the cohort population was approximately 6.9 and scores of 7 and 8 represented potential population means in response to an effective intervention strategy. In this way, binary and categorical exposure measures facilitated comparison with results from prevention and early intervention studies on adolescent mental health, which often report impacts as changes in the population mean on a symptom rating scale (e.g., standardized mean difference) or as relative risk of incidence or remission of clinically significant challenges.

**Table 1:** Variables included in the analysis.

Domain	Variables
**Exposure** ^a^	
Mental health	Score on Mental Health Inventory-5 psychological distress subscale
**Outcomes** ^**b**^	
Education	At least: high school, some college, college, more than college
Employment	Hours and weeks worked last year, any labor force participation
Income	Income from working last year, total assets at age 30 years
Health	Self-rated overall health, mental health, Medicaid/Medicare coverage
**Explanatory variables** ^**c**^	
Basic demographics	Age, month of birth, race, gender, region of the country, urbanicity
Neighborhood environment	Youth assessment of proportion of peers that smoke, engage in extracurriculars, will go to college, or engage in delinquency; youth and interviewer rating of home and neighborhood safety
Family resources	Family arrangement, household size, caregiver(s) income, parental education, parental self-rated health, whether ever experienced hard times, amount of government assistance, access to enriching activities, age of mother at first birth, age of mother at youth’s birth
Family processes	Indices of family routines; parental monitoring, limit setting, and breaking; and caregiver supportiveness; youth report of parenting styles
Physical health	Presence of childhood health conditions, birth defects, learning disabilities, or intellectual disabilities
School quality	Youth reports on teacher quality, classroom disruption, fair grading and discipline, and school safety
Academic skills	Grades in eighth grade, number of days doing homework on weekdays

^a^ Collected in round 4.

^b^ Collected in round 14, except for total assets, which was collected at age 30 years.

^c^ Collected in round 1, except for grades in eighth grade, which were collected in the round after which the individual had completed eighth grade.

#### Outcomes

We included 5 sets of outcome variables: labor supply, income, education, health, and health insurance coverage. Each of these variables was self-reported and captured in round 14, in 2010, 10 years after the exposure variable was captured, except for financial assets, which was captured at age 30 years for all participants. We selected a timeframe of 10 years because this is the same window over which many budget analysis entities, including the CBO, score the impacts of policies. We did not apply a discount rate throughout this study, as the goal is to estimate the actual economic impacts, rather than to assess the benefits in a decision context where considerations relevant to a discount rate are present, such as opportunity cost, time preference, or diminishing marginal utility [29]. All dollars were reported as 2022 USD, adjusted for inflation using CPI-U (Consumer Price Index–Urban).

Labor supply was measured in 3 ways: past-year labor force participation in the civilian labor force, number of weeks worked in any job in the past year in the civilian labor force, and number of hours worked in any job in the past year in the civilian labor force. Labor force participation in the civilian labor force was determined by whether an individual reported working any weeks at any job in the civilian labor force or reported looking for work. These metrics provide key inputs into the labor supply components of macroeconomic models.

Income included measures of both total self-reported income from working in any job in the civilian labor force in the past year and total self-reported financial assets at age 30 years. For both, respondents could either report a specific dollar amount or a range of dollar amounts. When respondents reported their income or assets as a range, we used the central point in the range as the actual value. The income component focused only on wages from employment rather than total income, which can be an input in several different pieces of a macroeconomic model. The assets are relevant to estimating changes in the capital supply.

For our education outcomes, educational attainment was recoded as a series of binary variables of at least high school completed, at least some college completed, at least college graduation, and more than college completed.

Health included self-rated overall health and psychological distress. Self-rated overall health was assessed using a single-item measure with levels of excellent, very good, good, fair, and poor [30]. Psychological distress was measured in the same way as the independent variable with the MHI-5, although treated as a continuous variable with scores from 0 to 9.

Health insurance was captured through a self-report of primary health insurance over the past year and was dichotomized between Medicaid/Medicare and any other coverage, including no insurance. The federal government would only pay directly for healthcare utilized while an individual is insured by Medicaid or Medicare (as a result of disability, given the young age of this population), so we separated out these insurance types. In models of the impact of investments in mental health on later public spending, the distinction between healthcare costs accrued under public health insurance coverage as opposed to private would be an important consideration.

#### Explanatory variables

To account for potential confounding in the relationship between adolescent psychological distress and later outcomes, we included explanatory variables in domains of basic demographics, neighborhood environment, family resources, family processes, physical health, school quality, and academic skills, driven by a theory of how mental health problems in adolescence relate to later labor outcomes and human capital formation more broadly [31]. NLSY97 round 1 measured our explanatory variables, when the most robust data in the cohort were collected. By using variables from 3 years prior to when the exposure variable was measured, we captured the developmental context for adolescents (e.g., the family arrangement they had during formative years) but not disruptions that may have occurred between rounds 1 and 4 (e.g., loss of a family member). These disruptions represent potential intervention targets that we may not want to control out of the final estimate (e.g., impacts on mental health and later outcomes from the loss of a family member could be moderated through interventions that focus on coping after bereavement).

Basic demographics included age, race and ethnicity, gender, region of the country, urbanicity, and month of birth. Race and ethnicity were coded together in the NLSY97 as black, Hispanic, mixed race (non-Hispanic), and non-black/non-Hispanic, based on caregiver report. While this coding missed different racial and ethnic interactions, it still provided valuable information about an individual’s racial and ethnic identity. Region of the country comprised 4 options based on census categories of Northeast, North Central, South, and West. Urbanicity allowed for 3 options: not in a metropolitan statistical area (MSA), in an MSA but not in the central city, and in an MSA and in the central city. We also included birth month to account for any effects that this may have on outcomes, such as the impacts of school entry at slightly different developmental stages [32].

Neighborhood environment included measures of peer behaviors and of neighborhood safety. The peer behavior variables asked the youth questions about “what percentage of kids in your grade…” and included smoking, engagement in extracurricular activities, plans to go to college, or engagement in delinquency. The neighborhood safety variable was an index that included questions that were both youth- and interviewer-reported on the availability of heat and electricity, quality of the buildings in the neighborhood, quality of the interior of the home, concerns for safety, and hearing gunshots. The basic demographic and neighborhood environment variables influenced exposures and access to opportunities across development that impact psychological distress or later economic outcomes, such as the effects of racism, community violence, or peer norms.

Family resources included variables that captured household circumstances and caregiver education, health, and income. The family arrangement variable identified the caregivers present in the youth’s life, including whether one or both biological parents were in the home or whether caregivers were adoptive, grandparents, or other individuals. Household size indicated the number of siblings and other individuals across whom resources (both financial and attentional) may be split. The age of the mother when she had her first child and the age of the mother when she had the respondent youth were also included, which offered information about resources for childrearing and birth order. Because some values of maternal birth age appeared nonsensically high or low, we recoded the variable into a factor with levels of younger than 18, 18–23, 24–29, and older than 30 to reduce bias from inaccurate responses. Caregiver(s) health was reported by a single respondent for both caregivers if they had a partner and was based on the same self-rated assessment of overall health as the health outcome variable. Caregiver(s) highest level of education was similarly reported by a single caregiver. In the NLSY97, the caregiver respondent was selected by first interviewing an individual over the age of 18 to create a household roster and then selecting a caregiver from the available adults based on a prioritized list of options, with biological mother being the first choice and a non-relative youth who acts as a father figure as a 13th choice.

Caregiver(s) (e.g., parent) income was computed based on questions about sources and amounts of annual income, as reported by a responding caregiver. As with the income-related outcome variables, if respondents reported the values as a range, we used the central point in the range. In calculating total annual income, we included income from farms or businesses, which could have negative values. When respondents reported income from farms or businesses as a loss but did not specify a value (which they were able to do if they reported income as a range), we used the median value of losses from respondents who did specify an amount. Caregivers also reported the number of months in the past year they received some form of government assistance, such as the Supplemental Nutrition Assistance Program, excluding unemployment insurance or worker’s compensation. Caregivers also answered whether the youth had experienced hard times growing up, such as having to “live in a place without water or electricity, or in a homeless shelter.” We also incorporated an index of whether the youth had an enriching environment, which included youth-reported questions on the presence of a computer in the home, a dictionary in the home, and engagement in extracurricular activities. These family resource variables captured exposures and access to opportunities and capacity for investment by adults in a child’s life, which is associated with both adolescent psychological distress and later economic outcomes.

Family process variables further built on the family resource variables by including the day-to-day developmental context for a child, which shapes a child’s psychological functioning as it relates to both psychological distress and later economic outcomes. An index of family routines measured the number of days each week the youth reported that they ate dinner with their family, housework got done, they did something fun as a family, or they did something religious as a family. We also included a youth-reported index of parental monitoring by any residential caregivers, which asked how much the caregiver knows about the youth’s close friends, the youth’s close friends’ parents, who the youth was with when not at home, and the youth’s teachers and school life. Both caregivers and youth reported on whether limits are set by caregivers, the youth, or jointly for how late the youth stayed out at night, who they could hang out with, and what media they were allowed to consume, and caregivers and youth also both reported on whether the youth broke these limits. Parenting style was coded as uninvolved, permissive, authoritarian, and authoritative, based on how youth answered 2 questions about how supportive and how strict each caregiver is. Two youth-reported variables captured the extent to which residential caregivers supported one another, if 2 residential caregivers are present, through a five-point Likert scale on how often each person in the relationship screamed when angry, compromised when in disagreement, expressed affection, insulted or criticized ideas, encouraged for important issues, or blamed for their problems.

A child’s other health challenges could have also caused mental health problems and impaired later health and economic success. We included variables on caregiver reports of the presence of different types of birth defects, chronic conditions (e.g., diabetes), learning disabilities, and intellectual disabilities. We did not include a variable of self-rated health for youth because evidence indicates that it also captures psychological distress, hence poor self-rated health can be thought of as the consequence of psychological distress [33].

We took school quality variables from four-point Likert scale measures about the extent to which a youth agrees with different statements. These statements included whether the teachers were good, whether the teachers were interested in the students, whether students disrupted learning, whether students were graded fairly, whether students cheated on tests, whether the discipline was fair, and whether the youth felt safe at school. These variables captured not only aspects of the quality of educational opportunities available to the child, but also the child’s sense of attachment to these opportunities, both of which have important implications for later development. Finally, we measured academic skills through a youth report of average grades in eighth grade—a time point several years before the exposure was measured when children are aged approximately 12 to 13 years. We coded grades in eighth grade as a factor with levels of mostly Cs and Ds (almost unsatisfactory or unsatisfactory performance), mostly Bs and Cs (satisfactory performance), mostly Bs (above average performance), and mostly As (exceptional performance). We also included a variable of youth-reported usual number of weeknights spent doing homework to incorporate a measure of motivation or caregiver influence on academic persistence. Academic skills, attitudes toward school, and psychological distress share an endogenous relationship with one another—psychological distress can impair academic success and school difficulties can promote psychological distress—while both relate to later health and economic outcomes [34]. Accounting for academic skills and attitudes toward school without teasing out the endogeneity may lead to an underestimation of the impact of psychological distress on later health and economic outcomes. We opted to completely account for academic skills and school attitudes, leading to a conservative underestimation of the psychological distress effects.

#### Analytic approach

We estimated the average treatment effect (ATE) of the exposure (presence or level of psychological distress) on the outcomes of employment, income, education, and health, accounting for the explanatory variables identified. The ATE is a causal contrast, comparing expected potential outcomes for our population of interest under 2 hypothetical states of the world: if everyone had past-month clinically significant psychological distress at one time point between ages 15 and 17 years, or if no one did, without regard for whether they received treatment or subsequently sought treatment. This can be extended to estimate contrasts between different levels of psychological distress. Although this study did not test the effects of a particular intervention, the results may bear on the potential impacts of a preventive intervention that reduces the risk of developing clinically significant psychological distress in adolescence.

We used targeted maximum likelihood estimation (TMLE) with machine learning algorithms to estimate the ATE [35–37]. This built on the developing literature on the possibility of generating causal evidence with observational data when certain identifying assumptions were met, particularly with the use of machine learning–enabled methods [38–40]. TMLE offered important advantages for addressing our research question with the available data. Traditional regression approaches, such as propensity score or G-computation methods, only produced unbiased results if the exposure or outcome mechanism was consistently estimated and the functional form was correctly specified. TMLE offered doubly robust estimation of an ATE, such that it would produce unbiased estimates if either the exposure mechanism or the outcome mechanism was consistently estimated, conferring an advantage over more traditional regression approaches. TMLE also accommodated machine learning techniques to minimize bias from misspecified regressions, which was critical here insofar as we had many covariates and were not certain of the correct functional form for the outcome and exposure models (e.g., nonlinearities and interactions among them).

TMLE allowed for a causal interpretation when 4 assumptions were met. First, the psychological distress of one individual must not have affected the outcome of others (noninterference), which was likely true for NLSY97 respondents from different households, but approximately 12% of the cohort were respondents from the same household. To examine the extent to which potential interference may have biased the results, we tested the impact of including just one individual per household selected at random in a sensitivity analysis. Second, the level of measured psychological distress exposure must have led to outcomes that arise from the given level of exposure (consistency) [41]. The MHI-5 offered a clinically validated dimensional measure of psychological distress that should have consistently captured the effects of psychological distress on cognitive, affective, and social functioning that impacted later outcomes across individuals [28]. Third, all confounders must have been measured (conditional exchangeability). We included direct or indirect measures of all potential confounders dictated by the theoretical framework, including 50 explanatory variables. We had only indirect measures of cognitive skills or personality, character, motivation, and aspiration, which included academic achievement in eighth grade, number of days doing homework on weekdays, interactions with family (e.g., rule-breaking or -following), and attitudes toward school and teachers. Potential existed for unobserved confounding that fell outside of the theoretical framework and poor measurement of identified confounders, which we discuss further in our limitations section. Finally, all clusters of individuals with different sets of values for the explanatory variables must have been able to develop (or not) psychological distress at all levels, which was true in our cohort because, although the different factors may modify the risk of developing psychological distress, none eliminate the risk completely (positivity).

With TMLE and machine learning algorithms that account for nonparametric and misspecified models, we calculated the ATE for all outcomes. We implemented TMLE in R version 4.0.5 using the *tmle* and *SuperLearner* packages, which applied machine learning approaches for the prediction steps to correct for misspecified regressions in TMLE, with 10 folds for cross-fold validation steps when the exposure was treated as a binary variable and 20 folds when the exposure was treated as a categorical variable, given the low variability in the exposure at some levels [42]. When implementing *SuperLearner*, we tested the *glm*, *gam*, *glmnet*, *ranger*, and *XGBoost* libraries, which applied generalized linear models, generalized additive models, elastic net regression, random forests, and extreme gradient boosting, respectively [43]. For comparison, we also ran TMLE with parametric model specifications and without machine learning algorithms. We did not implement corrections for multiple testing, as the focus was on ATE estimation rather than hypothesis testing, and we did not assess statistical significance. We did offer 95% confidence intervals (CIs), which should be interpreted cautiously given the lack of correction for multiple testing. All code and data used in the analysis are available in the supplement as items S1 Code and S1 Data, respectively.

*Missing Data*. We handled missing data in several ways. For all explanatory and outcome variables with missing data (which result from refusal to answer, uncertainty, invalid skips, or non-interview in the case of outcomes), we created a separate binary variable to capture that missingness, also known as a missingness indicator, and set all missing values to the median value (using the higher value in the case of categorical variables) [44]. Note that because some *SuperLearner* libraries cannot accommodate unordered factor variables, all unordered factors were recoded as a set of dummy variables, so identifying a median was not an issue (we rounded up when the median was between 2 values). Data missing because a caregiver was not present (e.g., residential father’s parenting style when there is no residential father) were accounted for through both the explanatory variable indicating missingness and through the explanatory variable indicating family arrangement. The variable for missingness in each outcome was passed into the TMLE function as a separate argument (“Delta”), whereas the missingness for each explanatory variable was passed as additional explanatory variables. This approach applies the machine learning capabilities of TMLE to incorporate information about the missing data, rather than relying on model-based specification and related assumptions about the mechanism, as with other common approaches to missing data [45]. For individuals who died by round 14, we coded all outcomes at the lowest level (0 for continuous, null for binary, and the lowest level of an ordered factor) rather than considering the outcomes missing as they otherwise would be in the NLSY97.

**Fig 1:**
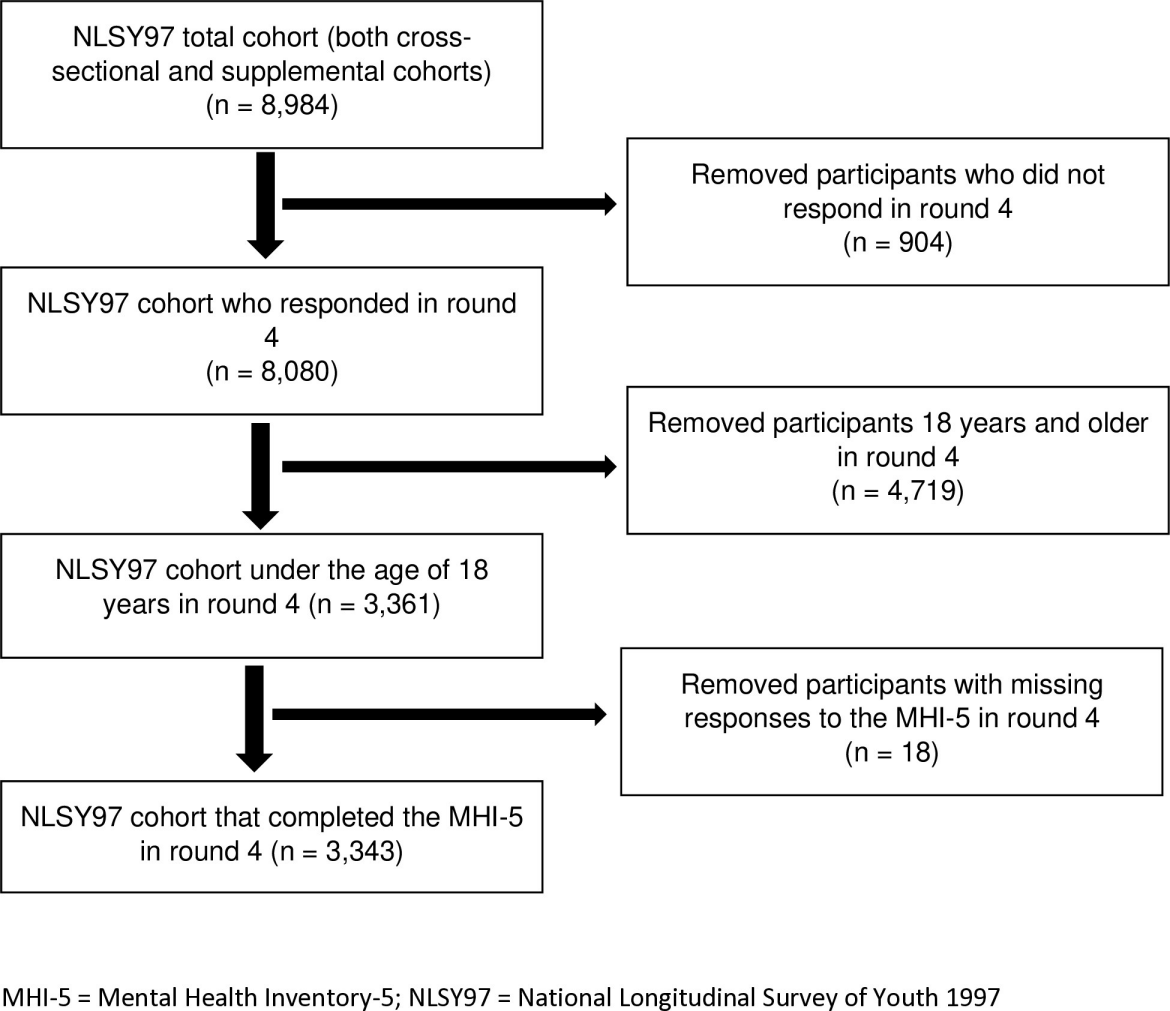
Cohort selection flowchart.

For the 18 cases missing exposure data, we excluded these individuals (see Fig 1 for cohort flowchart). Data were missing from the exposure when individuals refused to answer questions in the MHI-5 or responded that they were unsure. Some additional data were also missing because we only included those who were interviewed in round 4. Of the total NLSY97 (including those over the age of 17 years by round 4, who would not be part of our cohort), 10.1% of data was missing as a result of non-interview in round 4. Because each round was fielded over a period of several months (and an 18-month gap occurred between rounds 1 and 2), determining exactly what proportion of the cohort under a certain age could not be interviewed in round 4 is difficult. Approximately 41% of the cohort that was interviewed in round 4 was under age 18, so, if nonresponse was distributed evenly across the population, less than 5% of the total cohort would have been excluded from the subsample analyzed here.

#### Robustness checks

As noted, interference among siblings in the same household in our cohort may hinder causal inference. We tested the potential impacts of interference from inclusion of siblings in the same household in the cohort by repeating the analysis with siblings in the same household removed at random. We also accounted for possible bias introduced by our use of a subsample. To the extent that our choice to use the subsample of individuals who were interviewed and younger than 18 years during round 4 introduced selection bias that the measured explanatory variables do not account for, our ATE estimates from the subsample will not be reflective of the total sample (and thus the national population, given the representativeness of the NLSY97) [46]. To address this potential concern about the generalizability of the findings, we accounted for the use of a subsample by adapting an approach developed for TMLE [47]. We treated the outcomes for all individuals outside of the subsample (i.e., individuals older than age 17 in round 4) as missing, then calculated the population average treatment effects as contrasts between population mean outcomes at different levels of the exposure for the total sample. We also incorporated NLSY97 sampling weights to promote the generalizability of the estimates to the total US population in the analysis years. We calculated weighted standard deviations using provided sampling weights to determine the confidence intervals of the population average treatment effects, using the *Hmisc* package in R [48].

### Extending to hypothetical policy impacts on the federal budget

#### Data and sample

We drew data from several sources. The Current Population Survey (CPS), a program of the US Census Bureau and the Bureau of Labor Statistics that samples approximately 60,000 households each month, provided nationally representative data on employment and education in the United States at different ages and years. The National Survey on Drug Use and Health, a nationally representative survey administered by the US Substance Abuse and Mental Health Services Administration, asked youth whether they experienced a major depressive episode (MDE) in the past year. Our estimates of the relationship between adolescent psychological distress and later outcomes provided the remaining parameters. Our cohorts included the total populations reflected in the 2 surveys in 2022.

#### Approach to forecasting changes in labor supply over time

We estimated how prevalence of clinically significant adolescent psychological distress in 2022 would impact US labor supply growth for 2023 to 2032, holding other variables constant, using a simplified regression model:

$$LS_{a,t}=L{S^{o}}_{a,t}‐{\beta^{m}}_{a}m_{a}‐{\beta^{s}}_{a}(ED_{a}‐{S^{m}}_{a}m_{a}),$$

where *LS* represented the labor supply rate at age *a* and time *t*, *LS* ^o^_*a*,*t*_ represented the labor supply rate for those without clinically significant adolescent psychological distress at age *a* and time *t*, *β*^*m*^ represented the reduction in labor supply for those with adolescent psychological distress at age *a*, *m* represented the proportion of potential labor force participants of age *a* that experienced adolescent psychological distress, *β*^*s*^_*a*_ represented the reduction in the labor supply rate for those enrolled in education at age *a*, *ED*_*a*_ represented the educational enrollment rate of those age *a* without adolescent psychological distress, and *S*^*m*^_*a*_ represented the amount that the educational enrollment rate of those age *a* is reduced among adolescents with psychological distress.

We estimated *S*_*a*_ using the formula:

$$ENROLL_{a}=ED_{a}‐{S^{m}}_{a}m_{a},$$

where *ENROLL*_*a*_ was the proportion of individuals enrolled in education at age *a*.

We calculated *LS*_*a*,*t*_ based on the labor supply for people at each age *a* in the 2022 CPS data. We conducted 2 separate analyses, one that represented labor supply as any work in the past year and one that represented labor supply as the number of hours worked in the past year. For *m*_*a*_, we used data from the National Survey on Drug Use and Health on prevalence of past-year MDEs. While MDEs and the more general distress measured by the MHI-5 are not identical, this approach may have been able to offer a strong approximation, given that the MHI-5 is predictive of clinical depression. Note that, for simplicity, we did not allow the rate of psychological distress or the rate of enrollment in education to change with time. We calculated *β*^*s*^_*a*_ based on the difference in labor supply rates between those at age *a* not enrolled in education and those who are. *EDUC*_*a*_ was the proportion of individuals of age *a* in the CPS data in 2022 that reported being enrolled in education.

Our previous analysis supplied *β*^*m*^_*a*_ and *S*_*m*_. We used the ATE estimates from our analysis of the exposure as a binary variable. We used Monte Carlo analysis to capture the uncertainty associated with using multiple ATE estimates in a single model, drawing values from a normal distribution determined by the ATE mean and standard error and conducting 1,000 runs for a simulated cohort of 1,000 agents. Because our analysis only gave us an estimate of *β*^*m*^_*a*_ at a single time point, we tested 2 approaches: (1) holding *β*^*m*^_*a*_ constant at the same rate of labor supply impact; and (2) starting at half of the final value and incrementing consistently over the 10-year period. *LS*^*o*^_*a*,*t*_ and *ED*_*a*_ were then calculated based on these other values. For *S*_*m*_, we used the ATE for completing at least high school for age 18 years, completing at least some college for ages 19 and 20 years, completing at least college for ages 21 and 22 years, and completing more than college for ages beyond 22 years.

#### Budget impacts of a hypothetical policy

We then estimated how a hypothetical policy that altered the prevalence of clinically significant adolescent psychological distress would impact labor supply over 10 years, using the forecasting model. The hypothetical policy would fund the coverage and implementation of high-quality integrated mental health preventive interventions in primary care for adolescents [49,50]. Based on a recent meta-analysis, we estimated that the intervention decreased the relative risk of developing adolescent psychological distress by 0.71 (95% CI [0.51, 0.99]) (a potential overestimation because effect sizes in real-world implementation are often somewhat lower than those obtained in clinical trials) [49]. The preventive services reduced the total proportion of people with clinically significant adolescent psychological distress based on a Monte Carlo analysis that treated the distribution of relative risks from the previously cited meta-analysis as log-normally distributed. As our goal was only to test whether budget impacts were large enough to be worth systematically considering and not to evaluate a particular policy, we made some simplifying assumptions. We assumed that the policy was sufficiently funded to reach 10% of the adolescent population that would otherwise go on to develop depression, either through targeting with screening for risk factors or through broad-scale implementation, and that it was completely implemented in 2022. We did not consider the policy costs, as we focused on the magnitude of later budget effects, not cost-benefit analysis.

To estimate policy impacts, we contrasted baseline labor supply growth projections provided by the CBO for 2022 to 2032 with our projections under the scenario in which the hypothetical policy was implemented in 2022, reducing the prevalence of adolescent psychological distress and thus changing the forecasted trajectory of labor supply [51]. We assumed that the policy was completely implemented at *t* = 0 (2022 in this case), so that only those at *a = 18* would be impacted at *t* = 1 and so on until *t* = 10 (or 2032). To determine the total impact on labor supply growth at each *t*, we included all potential labor force participants between the ages of 18 and 64 years, including those who did not receive the intervention resulting from the policy, due to being older than 17 at the time of implementation.

The CBO published a workbook that allows users to examine how different scenarios—such as different growth rates in labor supply—would impact the budget, based on the CBO’s economic model. We used the contrast in predicted annual labor supply growth between baseline and intervention in the context of CBO’s workbook to estimate the effects of the hypothetical policy on the federal budget [51]. This study was reported as per the Consolidated Health Economic Evaluation Reporting Standards 2022 Statement, available in the S1 Checklist [52].

## Results

### Estimating the relationship between adolescent psychological distress and later outcomes

The NLSY97 subsample included 3,343 youths. See Fig 1 for the sample selection flowchart. Table 2 shows the distribution of key variables and missing data for this subsample. Among the youth in the cohort, 4.4% (146) met the criteria for clinically significant psychological distress in the past month. A large proportion of the cohort was black and Hispanic (47%) and had other health or developmental challenges (43%). Ten years later, 84% of the cohort had any past-year labor force participation with an average of 1,483 (standard deviation [SD]: 1,064) total hours worked. On average, they earned $27,908 (SD: 27,265 USD) in income annually and had total assets at age 30 years of $29,419 (SD: 62,453 USD). Three percent of the cohort demonstrated past-month clinically significant psychological distress 10 years after the exposure was measured (i.e., individuals experienced ongoing psychological distress or new onset) and 24% of the cohort graduated college by this time point. Only 1% of the cohort died during the relevant time period. Several variables had a high amount of missing data, including 1 explanatory variable and 2 outcome variables with missingness over 15%. Nineteen percent of caregiver income data was missing, in part due to a data collection error during the first round. Similarly, 19% of total assets at age 30 years data was missing due to similar issues.

**Table 2:** Description of cohort included in the analysis.

Variable	Value (mean and STD or %)	Percent missing
Total cohort size	3,343	N/A
**Explanatory variables in childhood** (measured in round 1, 1997)		
Age	16.47 (0.55)	0%
Race/ethnicity (% black and Hispanic)	47%	0%
Gender (% female)	49%	0%
Caregiver(s) income (2022 USD)	$75,899 ($69,421)	19%
Household size (number of household members)	4.61 (1.50)	0%
Family arrangement (% 2 biological parents)	52%	1%
Past-year receiving public assistance (%)	1%	1%
Grades in 8th grade (% mostly As and Bs or higher)^a^	39%	2%
Other health or developmental challenges^b^	43%	11%
**Exposure** (measured in round 4, 2000)		
Score on MHI-5 (0–9)	6.84 (1.68)	0%
Clinically significant symptoms (MHI-5 score ≤3)	4.4%	0%
**Outcomes in adulthood** (measured in round 14, 2010)		
Number of hours worked past year	1,483 (1,064)	8%
Number of weeks worked past year	36 (21)	6%
Any labor force participation last year (%)	84%	6%
Income earned past year (USD)	$27,908 (27,265)	13%
Total assets at age 30 years (USD)	$29,419 (62,453)	19%
Educational attainment (% college graduate)	24%	12%
Overall health (% fair/poor)	13%	12%
Mental health problems (% significant)	3%	15%
Medicaid/Medicare coverage past year	12%	12%
Death	1%	4%

^a^Eighth grade contains children aged approximately 12–13 years. Grades of As and Bs represent above average academic performance.

^b^This includes any reported chronic health condition such as diabetes, birth defects, learning disabilities, or intellectual disabilities.

MHI-5, Mental Health Inventory-5; STD, standard deviation; USD, US dollars.

Table 3 offers cohort means and standard deviations across outcomes, stratified by the presence of clinically significant psychological distress in adolescence (i.e., the population with an MHI-5 score greater than or equal to 4 and the population with an MHI-5 score less than or equal to 3), and the difference in means between the 2 populations. Those in the subsample with clinically significant psychological distress in adolescence experienced worse health and economic outcomes later in life than their counterparts in all dimensions. These included 295 fewer hours worked over the past year, $10,146 (USD) less income earned over the past year, a 10 percentage point lower high school graduation rate, and a 17 percentage point higher rate of Medicaid or Medicare coverage.

**Table 3:** Descriptive subsample means, standard deviations, and differences in means for the economic and health outcomes stratified by severity of mental health challenges in adolescence.

Outcome	Population with MHI-5 score ≥4^a^	Population with MHI-5 score ≤3	Difference in means^b^
Any labor force participation, year 10	0.85 (0.70)	0.77 (0.83)	−0.08
Number of hours worked, year 10	1,523 (1,995)	1,226 (2,216)	−295
Number of weeks worked, year 10	37.35 (40.52)	29.81 (44.65)	−7.48
Income earned, year 10 (USD)	$33,260 (59,570)	$23,114 (48,250)	−10,146
Total assets, age 30 (USD)	$25,020 (113,316)	$12,053 (81,381)	−12,967
Education (at least the amount of education completed by year 10)			
More than college	0.07 (0.50)	0.03 (0.36)	−0.03
College	0.22 (0.81)	0.14 (0.68)	−0.08
Some college	0.69 (0.91)	0.57 (0.97)	−0.12
High school	0.91 (0.57)	0.81 (0.77)	−0.1
General health, year 10^c^	2.27 (1.88)	2.59 (2.03)	0.32
Mental health problems, year 10^d^	7.38 (3.06)	6.51 (3.58)	−0.86
Medicaid/Medicare coverage, year 10	0.10 (0.59)	0.27 (0.88)	0.17

^a^ Population means and standard deviations for that subsample.

^b^ Simple difference in means between subsamples.

^c^ Higher values indicate worse self-reported health.

^d^ Lower values indicate worse mental health.

MHI-5, Mental Health Inventory-5; USD, US dollars.

Table 4 provides our estimates of the ATEs of clinically significant psychological distress in adolescence on later health and economic outcomes, implementing TMLE with machine learning algorithms. We found that the presence of past-month clinically significant psychological distress in adolescence may have led to impacts on employment outcomes, including a 6-percentage-point (95% CI [−0.08, −0.03]) reduction in any past-year labor force participation in the civilian labor force, 201 (95% CI [−259, −142]) fewer past-year hours worked, and 5.71 (95% CI [−7.18, −4.24]) fewer past-year weeks worked, 10 years later. We saw similar impacts in income, with $5,658 (95% CI [−6,772, −4,545] USD) fewer past-year dollars earned from wages at any job in the civilian labor force a decade later and $10,833 (95% CI [−15,042, −6,624]) fewer total financial assets at age 30. For education outcomes, clinically significant adolescent psychological distress had the greatest impact on completing at least some college—a 9-percentage-point (95% CI [−0.11, −0.07]) reduction—with declining impacts at each level of attainment thereafter. Finally, we found that clinically significant adolescent psychological distress led to an increase of 0.27 points (95% CI [0.21, 0.33]) on a five-point scale of self-rated health, with higher scores indicating worse health; a 0.93 point (95% CI [−1.05, −0.80]) decrease on a nine-point scale of psychological distress, with lower scores indicating worse mental health, and an 11-percentage-point (95% CI [0.08, 0.13]) increase in Medicare and Medicaid coverage 10 years later.

**Table 4:** Nonparametric estimates of effects of adolescent psychological distress as a binary variable on economic and health outcomes.

Outcome	Average treatment effect (95% CI)
Any labor force participation, year 10	−0.06 (−0.08, −0.03)
Number of hours worked, year 10	−201 (−259, −142)
Number of weeks worked, year 10	−5.71 (−7.18, −4.24)
Income earned, year 10 (USD)	−$5,658 (−6,772, −4,545)
Total assets, age 30 (USD)	−$10,833 (−15,042, −6,624)
Education (at least the amount of education completed by year 10)	
More than college	0.00 (−0.05, 0.06)
College	−0.03 (−0.04, −0.01)
Some college	−0.09 (−0.11, −0.07)
High school	−0.07 (−0.10, −0.04)
General health, year 10^a^	0.27 (0.21, 0.33)
Mental health problems, year 10^b^	−0.93 (−1.05, −0.80)
Medicaid/Medicare coverage, year 10	0.11 (0.08, 0.13)

*The average treatment effect reports the contrast between the population mean estimates if the entire cohort had clinically significant psychological distress in adolescence (MHI-5 ≤3 in round 4) or did not have clinically significant psychological distress in adolescence (MHI-5 ≥4 in round 4).

^a^Higher values indicate worse self-reported health.

^b^Lower values indicate worse mental health.

CI, confidence interval; MHI-5, Mental Health Inventory-5; USD, US dollars.

In S1 Text, we offer results from a parametric implementation (Table A in S1 Text), the extension where adolescent psychological distress is treated as a categorical variable (Tables B and C in S1 Text), and several checks on robustness (Tables D and E in S1 Text). The parametric implementation produced similar, although generally slightly smaller estimates across outcomes, but with greater variability. The categorical extension of the analysis found that changes across levels of adolescent psychological distress were associated with inconsistent changes in later outcomes, with greater variability in estimates. The robustness checks generally found similar results as the main analysis, with the impact of siblings from the same household likely being small and the impact of subsample selection leading to similar point estimates, although with large variability in the estimates. S1 Table presents a balance table: a list of standardized mean differences between explanatory variables for the groups with and without adolescent psychological distress, as further examined in S1 Text. S2, S3, and S4 Tables offer coefficients from the parametric implementation, including the Q model and g model, the 2 regressions used for doubly-robust estimation, as well as the Delta model, the regressions used for account for missing variables, as further explained in S1 Text.

### Extending to hypothetical policy impacts on the federal budget

Table 5 provides estimates of how a hypothetical policy expanding access to mental health preventive care and reaching 10% of youth who would otherwise go on to develop mental health challenges would affect annual growth in hours of labor supplied nationally each year and the associated estimated annual budget impacts. We found that the hypothetical policy would produce an approximately 0.02% change in annual growth in hours of labor supplied in many years between 2023 and 2032, potentially leading to a cumulative $52 (95% credible interval [CrI] [51,54] USD) billion in additional federal revenue over 10 years. The Supplemental Modeling Results in S1 Text, including Tables F and G in S1 Text, offer results from the sensitivity analyses. Analyzing the change in labor supply as any past-year labor force participation rather than past-year total hours worked decreased the estimated budget impacts. Modeling the impact of adolescent mental health as smaller in the early years and growing over time also decreased the estimated budget impacts, although both sensitivity analyses still produce cumulative benefits over $20 billion (USD).

**Table 5:** Estimated federal budget impacts from a hypothetical policy, based on effects on rate of past-year hours worked.

Year	Annual growth in past-year hours (%) (95% CrI)	Budget impact (billions of USD) (95% CrI)
2023	0.02 (0.02, 0.02)	$1 (1, 1)
2024	0.02 (0.02, 0.02)	$1 (1, 1)
2025	0.03 (0.03, 0.03)	$2 (2, 2)
2026	0.02 (0.02, 0.02)	$3 (3, 3)
2027	0.02 (0.02, 0.02)	$4 (4, 4)
2028	0.02 (0.02, 0.03)	$5 (5, 6)
2029	0.02 (0.02, 0.02)	$7 (7, 7)
2030	0.02 (0.02, 0.02)	$8 (8, 9)
2031	0.02 (0.02, 0.02)	$10 (10, 10)
2032	0.02 (0.02, 0.02)	$11 (11, 12)

CrI, credible interval; USD, US dollars.

## Discussion

Our results find that psychological distress during ages 15 to 17 years is related to a range of worse economic and health outcomes approximately a decade later. With limitations discussed as follows, the analysis offers a strong association and builds toward a potentially causal interpretation that further triangulation with other datasets can validate. Budget analysts can incorporate these results as parameters in existing economic models to estimate policy impacts. Our exploratory analysis with a hypothetical policy further indicates that, if a policy can produce population-level changes in the prevalence of clinically significant psychological distress, the policy may result in substantial budget impacts that may be important to consider systematically when producing budget forecasts.

Our findings align in magnitude with previous literature estimating the relationships between adolescent mental health and later economic and health outcomes [13–15]. In addition to offering a more comprehensive set of estimates, our study builds on this literature by leveraging innovative, machine learning–enabled methods that allow for doubly robust estimation of effects, correction for model misspecification, and fewer assumptions about mechanisms of missingness for outcome and confounder data. These estimates allow us to forecast some of the potential impacts of policies that improve adolescent psychological distress on the US macroeconomy with fewer issues of unmeasured confounding than previous studies [24].

Our findings are also relevant in a current policy context. In 2023, the US federal legislature passed a policy investing $60 million annually in integrated mental health care infrastructure [53]. Based on awarded grants, the program aims to expand access at a rate of approximately 500 individuals per $1 million, leveraging other recent changes to public and private insurance reimbursement for sustainability [54]. To reach 5 million people (roughly 25% of the adolescent population), the legislature would need to expand this program and invest at least $10 billion. Given the estimated savings of $52 billion over 10 years if the interventions can reach 10% of adolescents who would otherwise go on to develop depression, investments in adolescent mental health at scale will plausibly provide significant offsetting returns.

In general, while many important policies likely impact adolescent mental health, few policy efforts have targeted a specific amount of population-level change in the prevalence of adolescent mental health conditions. Policymakers need options that could plausibly yield these impacts to achieve offsetting returns. Aside from the hypothetical policy explored here, other policy strategies could also be effective. The legislature could focus on structurally embedding preventive interventions into the institutions that touch the lives of children, such as incorporating social and emotional skill-building in schools, early care, and community settings, to reinforce impacts at all levels [55]. Policy could mitigate systemic risk factors for adolescent mental health challenges, such as by providing cash transfers to reduce family stress and exposure to adversity [56,57]. Ideally, policy action should focus on the upstream drivers of declining mental health among adolescents, although additional research is needed to identify the most salient contributors and to determine effective policy solutions for addressing them [58].

Notably, our analysis only considers a single pathway for how changes in adolescent mental health impact the federal budget: effects on labor supply. The analysis does not consider effects on capital supply, productivity, or direct impacts on revenue or expenditures (which may be substantial, given the potential effects on coverage by public health insurance). With all pathways to budget impacts included, analyses may find that adolescent mental health policies produce budget impacts that even more substantially offset their costs.

Any successful policy strategy for achieving population-level impact on adolescent mental health would likely require a substantial outlay of resources, but population-level impact may also produce offsetting budget returns. If policymakers invested to enable the entities that conduct policy impact analyses to routinely estimate potential offsets, effective policies could face fewer unnecessary procedural barriers to enactment. With more effective policies in place, adolescents in the United States could experience greater wellbeing in the years to come.

This study has limitations. First, some data are missing. While we attempted to account for missing data in the explanatory variables and outcomes in the estimation procedure and the number of individuals excluded due to missingness in the exposure is small, missingness still introduces the possibility of bias into our estimates. Further, the use of a missingness indicator to handle missing data in the explanatory variables may introduce its own set of biases, although it likely offers less biased results than complete case analysis, and the assumptions for multiple imputations were not satisfied [59].

Second, we included measures for all potential confounders indicated by theory, but, to the extent that we did not include important confounders in our analysis or the measures used did not completely capture the confounding, this can also introduce bias. For example, we did not have direct measures of cognitive ability and motivation in rounds 1 through 4 but instead measured them indirectly with grades received in eighth grade and number of weekdays spent doing homework. This approach captures these constructs to some extent, but additional unmeasured confounding could be a source of bias.

Third, the effects estimated are for adolescents in the year 2000, and their outcomes in approximately 2010, and these estimates may not completely generalize to present day. For example, if educational and other institutions become more accommodating of mental health challenges, adolescent psychological distress may not lead to the same poor labor market and health outcomes. The outcomes were also measured during the Great Recession, and the findings may partially estimate the extent to which those who had adolescent psychological distress faced worse outcomes during downturns in the business cycle. The effects may be less pronounced during upswings in the business cycle, which also poses a limitation to our exploratory analysis of the potential long-term budget impacts of a hypothetical policy.

We also faced other limitations. The number of observations at certain levels of the exposure and of the outcomes, when treated as categorical variables, was small, which may increase the variance of those estimates. The presence of siblings may have violated the non-interference assumption of the identification strategy, but our sensitivity analyses indicate that the bias introduced from including siblings is likely to be small. Future research should triangulate ATE estimates through similar analyses using other data sources [60].

This paper only began to examine the extent of later budget impacts from a hypothetical policy. Future research can use the parameters in this paper for more rigorous policy evaluation. This could include determining intervention costs and modeling uncertainty around implementation. It could also capture more budget effects, such as those from increased productivity or decreased public benefit utilization. Future research could also incorporate more nonfinancial impacts relevant to policymakers, such as other public health, wellbeing, and societal benefits.

In conclusion, psychological distress during adolescence may lead to later economic and health consequences that are relevant to the US macroeconomy and federal budget within 10 years. If a policy could produce population-level reductions in adolescent psychological distress, it could potentially have substantial 10-year federal budget impacts. As explored, the parameters estimated in this paper can be incorporated into existing economic models to support policy impact analysis. By estimating the offsetting budget impacts of policies that address adolescent mental health, policymakers will be better equipped to appropriately respond to the growing adolescent mental health crisis in the United States.

## Supporting information

S1 Checklist Consolidated health economic evaluation reporting standards (CHEERS) checklist. (DOCX)

S1 Data Data. (CSV)

S1 Code Code. (RMD)

S1 Text Supporting information. (DOCX)

S1 Table Balance tables. (CSV)

S2 Table Q coefficients. (CSV)

S3 Table G coefficients. (CSV)

S4 Table D coefficients. (CSV)

## Abbreviations

ATE: average treatment effect
CBO: Congressional Budget Office
CI: confidence interval
CPS: Current Population Survey
CrI: credible interval
MDE: major depressive episode
MHI-5: Mental Health Inventory-5
MSA: metropolitan statistical area
SD: standard deviation
TMLE: targeted maximum likelihood estimation
