# Supplementary material for: Psychological distress in adolescence and later economic and health outcomes in the United States population: A retrospective and modeling study
Source: PLoS Med. 2025 Jan 16;22(1):e1004506. doi: 10.1371/journal.pmed.1004506 (PMC11737723; doi:10.1371/journal.pmed.1004506)
Supplement: S1 Text — (DOCX) [file pmed.1004506.s004.docx]

**S1 Text. Supporting Information**

**Psychological Distress in Adolescence and Later Economic and Health Outcomes in the U.S. Population**

Online-only Supplement

**Parametric Implementation**

Table A shows estimates of the effect of adolescent psychological distress on later health and economic outcomes when adolescent mental health is treated as a binary variable of clinically significant challenges and when targeted maximum likelihood estimation (TMLE) is implemented with parametric specifications and without machine learning algorithms. The point estimates for the relationship between mental health problems in adolescence and health and economic outcomes generally trend toward worse outcomes a decade later in this analysis but with high variability across all estimates. Unlike the unadjusted differences in population means across groups presented in Table 3 in the main paper, the average treatment effects (ATEs) estimated with the parametric implementation of TMLE that correct for confounding tend to be smaller.

To provide deeper insights into the analysis, S1 Table offers a balance table: the standardized mean differences between explanatory variables for the groups with and without adolescent psychological distress, before any processes were implemented to account for missing data and before any further analysis was conducted. Of the 171 explanatory variables (which includes a large number of dummy variables created for categorical variables), 21 variables had a standardized mean difference higher than 0.1, indicating poor balance in the unadjusted sample. Five covariates had a standardized mean difference higher than 0.2, including household income, mother being supportive, father being supportive, father monitoring of behavior, and gender.^[[1]](#footnote-2)^

S2 Table, S3 Table, and S4 Table offer the values of the coefficients from the parametric models used to estimate the number of hours worked in the past year in any job in the civilian labor force in this parametric implementation of TMLE. The first set of coefficients are from the Q model, which is the regression for obtaining initial estimates of the expected outcomes, based on the exposure and explanatory variables, before the “targeting” steps in TMLE. The second set of coefficients are for the g model, which is the regression for estimating the likelihood of receiving the exposure (clinically significant adolescent psychological distress), given the explanatory variables. The final is the coefficients for the Delta model, which is the regression used to estimate the likelihood of missingness in the outcome, given the exposure and explanatory variables.

The estimates from the nonparametric implementation in the main paper are likely to be less biased, and all demonstrate substantially less variability. In summary, our parametric analysis may have led to potential underestimates of some employment, earnings, and health effects and potential overestimates of some education variables.

**Categorical Extension**

We then examine the ATEs for adolescent mental health as a categorical variable, based on contrasts among reasonable population mean Mental Health Inventory-5 (MHI-5) scores of 6, 7, and 8. Table B offers the unadjusted means and standard deviations for economic and health outcomes 10 years later for the individuals in the sample with these scores in round 4. At this low level of reported symptoms, reporting fewer symptoms (i.e., higher MHI-5 score) is not consistently associated with worse long-term outcomes. Those who scored a 7 on the MHI-5 often had better employment, earnings, and educational outcomes on average than those who scored an 8. Only the general health and mental health variables demonstrated the expected progression, in which those with worse adolescent mental health experienced worse later general health and mental health on average.

Table C shows the results from the nonparametric implementation of TMLE with the exposure as a categorical variable. This represents a series of hypothetical scenarios in which everyone in the population had a specific MHI-5 score in adolescence and then reports the contrasts among them. After correcting for confounding, the analysis still finds potentially counterintuitive results. As the population mean score increases, indicating improvements in mental health, labor force participation, income earned, graduating more than high school, graduating at least high school, and having Medicaid or Medicare coverage all demonstrate inconsistent average changes across levels. Total assets at age 30, graduating at least college, general health, and mental health all improve on average with improving population adolescent mental health. Unexpectedly, however, number of hours worked, number of weeks worked, and graduating at least some college showed consistent declines on average with improving adolescent mental health. Although this study did not focus on hypothesis testing, it still should be noted that almost all of the 95% confidence intervals across all estimates included zero. The potential lack of significance would be even more pronounced after correcting for multiple hypothesis testing, so particular caution should be taken when analyzing these results.

Several potential issues could explain the counterintuitive findings for the exposure as a categorical variable. First, the MHI-5 may be able to detect the presence of clinically significant psychological distress but not demonstrate sufficient sensitivity at low levels of symptomatology to discriminate among degrees of need. However, the analysis may also be measuring a real phenomenon. Slight adversity in adolescence, which could manifest as poor mental health symptoms (i.e., working hard at school but feeling stressed), could help to build resilience and promote better employment, economic, and educational outcomes later in life. The analysis may have also failed to completely account for confounding from differential cognitive endowments, which could be associated with slightly elevated (but not clinically significant) mental health problems in adolescence. As discussed later, these possibilities point to the need for triangulation of ATEs through follow-up studies with other datasets.

**Robustness Checks**

To explore the impacts of potential interference from having siblings from the same household in the sample, Table D shows the nonparametric TMLE results when we only include one individual per household in the analysis, selected at random. This analysis reduced our sample size to 3,137 individuals. All estimates are within the 95% confidence intervals of the estimates from the full sample, and many of the estimates are qualitatively extremely similar. The specific ATEs vary slightly depending on which household member is randomly selected to be in the subsample. In this subsample, the deleterious effect of adolescent psychological distress on later outcomes is slightly less for hours worked, weeks worked, income earned, and later mental health problems. From this, we conclude that some risk exists for overestimation of effects on some outcomes, but that including siblings in the same household in our subsample likely did not contribute to substantial bias in ATE estimates as a result of interference.

We also repeat our analysis with the analytic strategy that accounts for potential selection bias in the use of a subsample and incorporates sampling weights, with findings reported in Table E. This allows us to obtain a population average treatment effect (PATE) for the United States. Due to the low variability in the outcome of those who completed more than college (i.e., most people did not complete more than college) and the nature of this analysis, which seeks to transport subsample ATE estimates to the larger sample and treats outcomes from the larger sample as missing, we were not able to estimate a PATE for this outcome. Many of the PATEs are similar to the ATEs estimated in the population subsample but with extremely high variability, which likely results from attempting to estimate outcomes across a full sample that is much larger than the subsample. If selection bias resulted from our use of a subsample, further research with larger subsamples will be necessary to estimate PATEs for adolescent psychological distress and later health and economic outcomes with less variability.

**Supplemental Modeling Results**

Table F shows the results of our sensitivity analysis in which labor supply is represented as any work in the past year and in which the impacts of adolescent psychological distress are at the same level all 10 years. The estimates find that the hypothetical policy could lead to $24 (95% credible interval: 24, 24 2022USD) billion in federal budget impacts over 10 years as a result of changes in labor supply. Table H offers the sensitivity analysis in which the impacts of adolescent psychological distress start out smaller and grow as individuals age. In this model, the effects decreased and the total budget impact is $49 (47, 49 2022USD) billion over 10 years.

**Table A. Parametric Estimates of Effects of Adolescent Psychological Distress as a Binary Variable on Economic and Health Outcomes**

| **Outcome** | **Average treatment effect (95% CI)** |
| --- | --- |
| Any labor force participation, year 10 | -0.05 (-0.31, 0.21) |
| Number of hours worked, year 10 | -161 (-831, 509) |
| Number of weeks worked, year 10 | -6.17 (-19.63, 7.29) |
| Income earned, year 10 (2022 USD) | -4,598 (-16,981, 7,784) |
| Total assets, age 30 (2022 USD) | -9,824 (-35,630, 15,980) |
| Education (at least the amount of education completed by year 10) | |
| More than college | -0.04 (-0.19, 0.12) |
| College | -0.03 (-0.22, 0.15) |
| Some college | -0.11 (-0.36, 0.15) |
| High school | -0.06 (-0.28, 0.17) |
| General health, year 10^a^ | 0.19 (-0.48, 0.86) |
| Mental health problems, year 10^b^ | -0.89 (-1.98, 0.21) |
| Medicaid/Medicare coverage, year 10 | 0.10 (-0.16, 0.37) |

^a^ Higher values indicate worse self-reported health

^b^ Lower values indicate worse mental health

CI = confidence interval; USD = U.S. dollars

**Table B. Unadjusted Means and Standard Deviations for Economic and Health Outcomes at Different Levels of Psychological Distress in Adolescence**

| **Value of interest** | **Score on Mental Health Indicator-5** | | |
| --- | --- | --- | --- |
|  | 6 | 7 | 8 |
| Sample size | 631 | 845 | 771 |
| Any labor force participation, year 10 | 0.86 (0.69) | 0.87 (0.67) | 0.83 (0.73) |
| Number of hours worked, year 10 | 1,476 (1,935.63) | 1,554 (2,050) | 1,522 (2,147) |
| Number of weeks worked, year 10 | 37.26 (39.66) | 38.02 (39.71) | 36.53 (41.74) |
| Income earned, year 10 (2022 USD) | 25,216 (45,596) | 30,717 (59,349) | 30,258 (54,014) |
| Total assets, age 30 (2022 USD) | 25,197 (104,114) | 32,607 (128,600) | 31,805 (122,800) |
| Education attained (at least the amount of education completed by year 10) | | | |
| More than college | 0.07 (0.52) | 0.09 (0.57) | 0.09 (0.56) |
| College | 0.23 (0.83) | 0.30 (0.90) | 0.28 (0.88) |
| Some college | 0.63 (0.95) | 0.72 (0.88) | 0.69 (0.91) |
| High school | 0.88 (0.63) | 0.92 (0.52) | 0.92 (0.53) |
| General health, year 10^a^ | 2.44 (2.01) | 2.30 (1.90) | 2.18 (1.88) |
| Mental health problems, year 10^b^ | 7.01 (3.14) | 7.29 (3.01) | 7.54 (2.90) |
| Medicaid/Medicare coverage, year 10 | 0.14 (0.67) | 0.10 (0.58) | 0.09 (0.57) |
| % Missing outcome data (weeks worked) | 6.6% | 5.2% | 4.9% |

^a^ Higher values indicate worse self-reported health

^b^ Lower values indicate worse mental health

USD = U.S. dollars

**Table C. Nonparametric Population Mean Outcome Estimates and Causal Contrasts of Adolescent Psychological Distress as a Categorical Variable for Economic and Health Outcomes**

| **Outcome** | **Score on Mental Health Indicator-5** | | | **Contrast among scores** | |
| --- | --- | --- | --- | --- | --- |
|  | 6 | 7 | 8 | 6 to 7 | 7 to 8 |
| Any labor force participation, year 10 | 0.83 (0.68, 0.99) | 0.86 (0.83, 0.88) | 0.81 (0.69, 0.92) | 0.02 (-0.12, 0.17) | -0.05 (-0.15, 0.05) |
| Number of hours worked, year 10 | 1,619  (1,040, 2,198) | 1,584  (1,177, 1,991) | 1,572  (1,213, 1,932) | -35  (-521, 450) | -11.92  (-389.99, 366.16) |
| Number of weeks worked, year 10 | 36.95  (27.49, 46.42) | 36.38  (29.62, 43.14) | 35.31  (27.39, 43.22) | -0.57  (-8.19, 7.05) | -1.07  (-7.79, 5.64) |
| Income earned, year 10 (2022 USD) | 25,464 (12,153, 38,777) | 29,461 (12,964, 45,955) | 29,035 (11,386, 46,687) | 3,996 (-7,534, 15,526) | -424  (-13,520, 12,672) |
| Total assets, age 30 (2022 USD) | 25,897 (-1,188, 52,983) | 29,758 (-11,042, 70,559) | 31,226 (-14,414, 76,867) | 3,862 (-21,808, 29,530) | 1,467 (-23,147, 26,082) |
| Education (at least the amount of education completed by year 10) | | | | | |
| More than college | 0.08 (-0.13, 0.29) | 0.07 (-0.08, 0.22) | 0.09 (-0.06, 0.24) | -0.01 (-0.14, 0.12) | 0.02 (-0.10, 0.13) |
| College | 0.23 (-0.23, 0.69) | 0.26 (-0.15, 0.66) | 0.28 (-0.12, 0.67) | 0.03 (-0.18, 0.23) | 0.02 (-0.14, 0.18) |
| Some college | 0.67 (0.29, 1.05) | 0.65 (0.19, 1.12) | 0.50 (-0.05, 1.05) | -0.02 (-0.33, 0.29) | -0.16 (-0.65, 0.34) |
| High school | 0.87 (0.70, 1.04) | 0.90 (0.75, 1.04) | 0.85 (0.65, 1.04) | 0.03 (-0.10, 0.15) | -0.05 (-0.22, 0.12) |
| General health, year 10^a^ | 2.42 (1.98, 2.85) | 2.34 (1.88, 2.80) | 2.19 (1.72, 2.67) | -0.08 (-0.48, 0.33) | -0.14 (-0.52, 0.24) |
| Mental health problems, year 10^b^ | 7.01 (6.91, 7.10) | 7.30 (7.09, 7.51) | 7.46 (6.90, 8.01) | 0.29 (0.06, 0.52) | 0.15 (-0.43, 0.73) |
| Medicaid/Medicare coverage, year 10 | 0.12 (-0.05, 0.30) | 0.11 (-0.04, 0.26) | 0.16 (-0.05, 0.37) | -0.01 (-0.17, 0.15) | 0.05 (-0.14, 0.24) |

^a^ Higher values indicate worse self-reported health

^b^ Lower values indicate worse mental health

USD = U.S. dollars

**Table D. Nonparametric Estimates of Effects of Adolescent Psychological Distress as a Binary Variable on Economic and Health Outcomes with Siblings Removed**

| **Outcome** | **Average treatment effect (95% CI)** |
| --- | --- |
| Any labor force participation, year 10 | -0.06 (-0.09, -0.04) |
| Number of hours worked, year 10 | -176 (-231, -121) |
| Number of weeks worked, year 10 | -5.03 (-6.48, -3.59) |
| Income earned, year 10 (2022 USD) | -5,006 (-6,060, -3,951) |
| Total assets, age 30 (2022 USD) | -10,700 (-13,703, -7,698) |
| Education (at least the amount of education completed by year 10) | |
| More than college | 0.00 (-0.04, 0.03) |
| College | -0.03 (-0.04, -0.01) |
| Some college | -0.09 (-0.11, -0.07) |
| High school | -0.07 (-0.09, -0.05) |
| General health, year 10^a^ | 0.30 (0.21, 0.39) |
| Mental health problems, year 10^b^ | -0.73 (-0.95, -0.51) |
| Medicaid/Medicare coverage, year 10 | 0.11 (0.09, 0.13) |

^a^ Higher values indicate worse self-reported health

^b^ Lower values indicate worse mental health

CI = confidence interval; USD = U.S. dollars

**Table E. Nonparametric Estimates of Effects of Adolescent Psychological Distress as a Binary Variable on Economic and Health Outcomes with Sampling Weights**

| **Outcome** | **Average treatment effect (95% CI)** |
| --- | --- |
| Any labor force participation, year 10 | -0.05 (-0.20, 0.10) |
| Number of hours worked, year 10 | -144 (-1,004, 716) |
| Number of weeks worked, year 10 | -4.46 (-12.97, 4.05) |
| Income earned, year 10 (2022 USD) | -5,772 (-19,127, 7,582) |
| Total assets, age 30 (2022 USD) | -15,690 (-51,396, 20,015) |
| Education (at least the amount of education completed by year 10) | |
| More than college | - |
| College | -0.04 (-0.32, 0.25) |
| Some college | -0.06 (-0.33, 0.22) |
| High school | -0.07 (-0.27, 0.13) |
| General health, year 10^a^ | 0.35 (-0.16, 0.85) |
| Mental health problems, year 10^b^ | -1.09 (-1.75, -0.42) |
| Medicaid/Medicare coverage, year 10 | 0.21 (-0.08, 0.50) |

^a^ Higher values indicate worse self-reported health

b Lower values indicate worse mental health

CI = confidence interval; USD = U.S. dollars

**Table F. Sensitivity Analysis of Federal Budget Impacts from a Hypothetical Policy, Based on Effects on Rate of Any Past-Year Work, with Stable Mental Health Effects**

| Year | Annual growth in past-year work (%)  (95% credible interval) | Budget impact (billions of 2022 USD) (95% credible interval) |
| --- | --- | --- |
| 2023 | 0.01 (0.10, 0.01) | 0 (0, 0) |
| 2024 | 0.01 (0.10, 0.01) | 1 (1, 1) |
| 2025 | 0.01 (0.10, 0.01) | 1 (1, 1) |
| 2026 | 0.01 (0.10, 0.01) | 1 (1, 1) |
| 2027 | 0.01 (0.10, 0.01) | 2 (2, 2) |
| 2028 | 0.01 (0.10, 0.01) | 2 (2, 2) |
| 2029 | 0.01 (0.10, 0.01) | 3 (3, 3) |
| 2030 | 0.01 (0.10, 0.01) | 4 (4, 4) |
| 2031 | 0.01 (0.10, 0.01) | 5 (5, 5) |
| 2032 | 0.01 (0.10, 0.01) | 5 (5, 5) |

USD = U.S. dollars

**Table G. Sensitivity Analysis of Federal Budget Impacts from a Hypothetical Policy, Based on Effects on Total Past-Year Hours Worked, with Lower Mental Health Effects**

| Year | Annual growth in past-year hours (%)  (95% credible interval) | Budget impact (billions of 2022 USD)  (95% credible interval) |
| --- | --- | --- |
| 2023 | 0.01 (0.01, 0.01) | 0 (0, 0) |
| 2024 | 0.02 (0.01, 0.02) | 1 (1, 1) |
| 2025 | 0.02 (0.02, 0.02) | 2 (2, 2) |
| 2026 | 0.02 (0.02, 0.02) | 3 (3, 3) |
| 2027 | 0.02 (0.02, 0.02) | 4 (4, 4) |
| 2028 | 0.02 (0.02, 0.02) | 5 (5, 5) |
| 2029 | 0.02 (0.02, 0.02) | 6 (6, 6) |
| 2030 | 0.02 (0.02, 0.02) | 8 (7, 8) |
| 2031 | 0.02 (0.02, 0.02) | 9 (9, 9) |
| 2032 | 0.02 (0.02, 0.02) | 11 (10, 11) |

USD = U.S. dollars

1. A balance table after further processing is not provided, because the use of the missingness indicator produces results that are difficult to interpret. [↑](#footnote-ref-2)
